# Reviewed article: Pereira, MIM; et al. American tegumentary leishmaniasis: epidemiological analysis and time series, Minas Gerais, Brazil, 2008-2024. Epidemiol Serv Saude. 2026:35;e20251212

**DOI:** 10.1590/S2237-96222026v35e20251212.a

**Published:** 2026-11-09

**Authors:** Dalila de Carvalho Silva Gonzaga

**Affiliations:** 1 Universidade de Brasília, Brasília, DF, Brazil Universidade de Brasília Brasília DF Brazil

## Peer review A

### Questionnaire

Does the manuscript contain new and significant information to justify publication? Yes 

Does the Abstract (Summary) clearly and accurately describe the content of the article? Yes 

Is the problem significant and concisely stated? Yes 

Are the methods described comprehensively? Yes 

Are the interpretations and conclusions justified by the results? Yes 

Is adequate reference made to other work in the field? No

### Manuscript Structure:

Length of article is: Adequate

Number of tables is: Adequate

Number of figures is: Adequate

### Rating

Interest Average

Quality Average

Originality Average

Overall Average

Recommendation: **Major Revision**

## Comments

Prezado autor,

O artigo precisa de alguns ajustes para ser publicados. Esses ajustes são de acordo com as normas da revista ou para enriquecer o manuscrito com informações relevantes da doença.

Sugestão de título:

Leishmaniose tegumentar americana no norte de Minas Gerais: análise epidemiológica e série temporal, 2008-2024

Aspectos éticos:

Utilizar a frase padrão da revista que é: Esta pesquisa utilizou bancos de dados de domínio público e anonimizados. Retirar os aspectos éticos antes do resultado. É NECESSÁRIO APENAS O ASPECTOS ÉTICOS DEPOIS DO RESUMO.

Introdução:

Primeiro parágrafo rever a seguinte frase: sendo apresentada com ampla distribuição no Brasil e no território brasileiro, configurando-se como um relevante desafio para a saúde pública e elevada carga de morbidade. Existe uma redundância na frase. Escrever de forma mais completa e simples.

Inserir na introdução mais informações sobre o protozoário do gênero Leishmania.

Fazer uma breve explicação sobre os flebotomíneos com o propósito de enriquecer o texto com mais informações.

Inserir também mais sintomas, diagnóstico e tratamento da doença.

Métodos:

Inserir após o Delineamento do estudo um tópico de Contexto. Neste tópico contexto escrever sobre as dimensões e população do Norte de Minas e citar as macrorregiões ou cidades do norte de Minas. Qualquer dúvida, verificar em artigos já publicados na RESS. Segue o link de um exemplo de artigo: https://www.scielo.br/j/ress/a/NrG84stwrV85tjGs6jr7kgQ/?format=pdf&lang=pt

Variáveis de estudos:

Citar apenas as variáveis da seguinte forma: raça/cor da pele, sexo, faixa etária e região de residência.

As variáveis clínicas incluíram evolução do caso, critério de confirmação, forma clínica, ocorrência autóctone no município de residência e tipo de entrada.

Porque a categoria “ignorado/branco” foi excluída de todas as variáveis?

Essa categoria é importante para verificar a incompletude dos dados. Além disso, implica na análise das tendências de incidência e na identificação de padrões de persistência e vulnerabilidade. Assim, poderia destacar na discussão o quão importante é preencher a ficha de notificação da doença corretamente pelo profissional de saúde.

Resultados:

O que significa os números na tabela 1? Seria algum código dos municípios? Se sim, por favor inserir uma coluna na tabela com o nome código do município.

Agrupar as faixas etárias em menos categorias para facilitar a compreensão do leitor

Discussão:

Faltam dados de estudos atuais no Brasil e no mundo sobre a Leishmaniose tegumentar americana. A discussão precisa ser mais ampla. Comparar os resultados do trabalho com outros estudos em outras regiões do país ou até mesmo de outras regiões de Minas Gerais. Utilizar dados internacionais de países endêmico e utilizar estudos atuais.

Discutir melhor a implementação de políticas públicas para combater/prevenir a doença ou os flebotomíneos.

Melhorar o último parágrafo da discussão de uma forma que esclareça a importância de desenvolvimento econômico e social, e políticas públicas que ajudem a combater as regiões endêmicas.

